# Real-world retention rate of secukinumab in patients with axial spondyloarthritis: Multi-centric retrospective data from China

**DOI:** 10.1515/rir-2026-0010

**Published:** 2026-07-13

**Authors:** Zhongchao Fu, Lin Wen, Ting Wu, Xinpeng Chen, Xiaoyu Liu, Sishi Huang, Lijun Zhang, Rangeng Shi, Xiaogui Cheng, Jinxian Huang

**Affiliations:** Division of Rheumatology, Department of Medicine, The Third Affiliated Hospital of Shenzhen University, Shenzhen, Guangdong Province, China; Division of Rheumatology, Department of Medicine, Ji’an Central People’s Hospital, Ji’an, Jiangxi Province, China; Division of Rheumatology, Department of Medicine, Shenzhen Futian Hospital for Rheumatic Diseases, Shenzhen, Guangdong Province, China; Division of Rheumatology, Department of Medicine, The University of Hong Kong-Shenzhen Hospital, Shenzhen, Guangdong Province, China; ShenZhen University Medical School, Shenzhen, Guangdong Province, China; Department of Pharmacy, The Third Affiliated Hospital of Shenzhen University, Shenzhen, Guangdong Province, China

**Keywords:** axial spondyloarthritis, secukinumab, retention rate, switching, discontinuation pattern

## Abstract

**Background and Objective:**

Secukinumab (SEC), an interleukin 17A inhibitors (IL-17Ai) has been shown to be effective in managing axial spondyloarthritis (axSpA). However, data on its long-term retention in Chinese populations remain limited. This study aims to evaluate the retention rate of SEC in axSpA and its predisposing factors in Chinese patients.

**Methods:**

We conducted a retrospective cohort study across three centers, including axSpA patients treated with SEC. Prescription records were retrieved from the hospital information system (HIS) and electronic medical records were reviewed to confirm diagnoses and treatment exposures. Retention rates at 12, 24, and 36 months were estimated using Kaplan-Meier analysis. Cox proportional hazards regression assessed the impact of prior biologic therapy and dosage on discontinuation risk. Discontinuation events were also evaluated.

**Result:**

A total of 459 patients were identified and included, with the longest treatment follow-up period reaching 42 months. The overall drug retention rates for SEC were 76.1%, 68.7%, and 62.8% at 12, 24, and 36 months, respectively. Biologics-naïve patients exhibited a higher 12-month retention rate (80.7%) compared to biologic-experienced patients (72.5%). Among biologic-experienced patients, the 12-month retention rates progressively decreased with an increasing number of prior biologics: 74.6% for one prior biologic, 72.0% for two, and 55.3% for three. No statistically significant difference in retention rate was observed between the 300 mg and 150 mg groups (*P* = 0.334). A total of 82 patients (17.86%) discontinued SEC. The primary reason for discontinuation was switching to alternative therapies (*n* = 70, 85.4% of discontinuations).

**Conclusion:**

SEC demonstrates robust retention rates in this Chinese axSpA cohort, particularly among biologics-naïve patients.

## Introduction

AxSpA is a chronic inflammatory disease that primarily affects the axial skeleton, including the spine and sacroiliac joints. The hallmark symptom of axSpA is chronic back pain.^[1]^ The exact cause is not fully understood, but it is believed that IL-17A plays a crucial role in the pathogenesis of axSpA.

Secukinumab (SEC), a fully human monoclonal antibody targeting and neutralizing IL-17A, has become an important treatment option for axSpA patients, especially those inadequately responding to conventional therapies.^[2]^

The retention rate of a medication reflects its effectiveness and tolerability in real-world settings and is influenced by factors such as efficacy, safety, patient satisfaction, and external elements like cost and accessibility. For SEC, data from open-label follow-up were available for only 5 years as a second-line treatment. Retention rates have been studied in both clinical trials and real-world settings. Real world evidence by FORSY cohort from French with 3 years observation of 906 cases revealed retention rate of 59% at the first year, with gender, body weight and biologic experience difference.^[3]^ Nonetheless, a small cohort of 60 cases from Poland reported retention rate of 98% at 1 year.^[4]^ No data from Chinese has been published. Therefore, we aimed to evaluate the retention rate of SEC in axSpA and its predisposing factors in Chinese patients.

## Method

### Study Design

**Figure 1:**
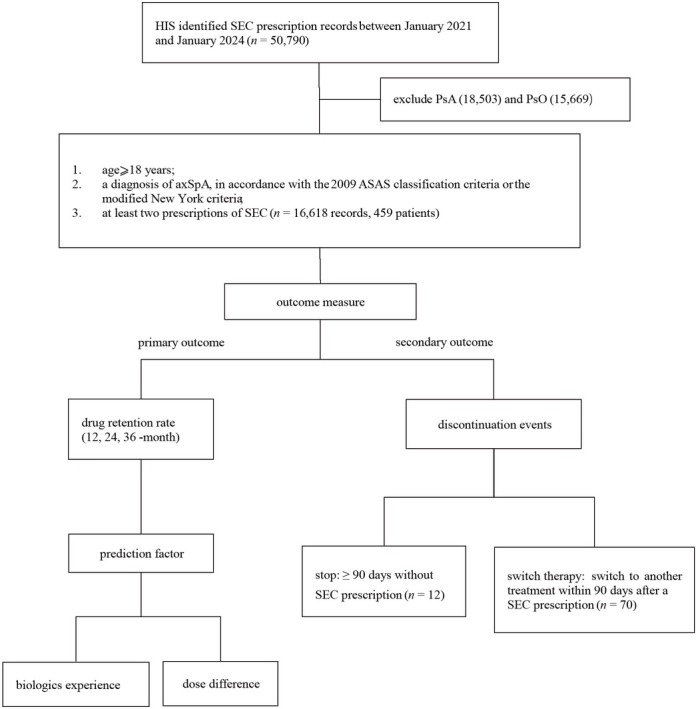
The flowchart of the study.

This is a retrospective study involving three centers with data sourced from the HIS and electronic medical record systems of the University of Hong Kong-Shenzhen Hospital, Shenzhen Futian Hospital for Rheumatic Disease and the Third Affiliated Hospital of Shenzhen University. The Ethics Committee’s approval was obtained from all participating centers (IRB No. 2024-LHQRMYY-KYLL-034; hkuszh2023081; FS202401001). Written informed consent for participation was not required for this study. Inclusion criteria were: age ≥ 18 years; a diagnosis of axSpA, in accordance with the 2009 ASAS classification criteria or the modified New York criteria,^[5,6]^ Initiation of SEC treatment between January 2021 and January 2024. Patients with at least two SEC prescriptions within 90 days were included. Those diagnosed with Psoriatic arthritis (PsA) and psoriasis (PsO) were excluded. The SEC initial loading or escalation dosage (150 mg or 300 mg) was determined by the treating rheumatologists based on routine clinical practice. This determination was primarily guided by factors such as the patient’s disease status (*e.g*. treatment refractory characteristics including very high disease activity, serum C-reactive protein [CRP] level and other objective signs of inflammatory activity indicated by imaging despite optimal treatment, concomitant extra-musculoskeletal manifestations such as psoriasis), and prior treatment history, consistent with the routine clinical indications for SEC use (BASDAI ≥ 4 or Ankylosing Spondylitis Disease Activity Score (ASDAS) ≥ 2.1 at the first visit). (A specific note: For patients who were already using SEC upon their first visit to our center, we did not immediately adjust their initial treatment regimen.) In our cohort, the standard initial regimen for eligible patients was 150 mg. The subsequent treatment strategy followed a predefined protocol: Dose escalation from150 mg to 300 mg was considered if the patient’s ASDAS score was ≥ 1.3 after approximately three months of treatment. Drug Discontinuation: If the patient’s ASDAS score remained ≥ 2.1 and ΔASDAS ≥ 1.1 was not met after a further three months on the intensified 300 mg dose, the treating physician would consider switching the patient to an alternative therapy. The final decision regarding initial dose selection and subsequent dose adjustment was at the discretion of the treating physician and shared decision making with the patient. The primary outcome observed was drug retention rate, and its predictive factors were analyzed. Secondary endpoints encompassed discontinuation patterns (Figure 1).

### Date Collection

Data collection focused on standard demographic parameters: age, sex, Body Mass Index (BMI), smoking history, and comorbidities, along with disease-related variables, including disease duration, Human Leukocyte Antigen B27 (HLA-B27) status, radiographic status, and extra-articular manifestations such as uveitis (UV), Psoriasis (PSO), and inflammatory bowel disease (IBD). Inflammatory markers, such as CRP and erythrocyte sedimentation rate (ESR). Imaging studies, including X-rays and Magnetic Resonance Imaging (MRI) of the sacroiliac joint (SIJ), were reviewed as clinically indicated. Treatment characteristics included previous biologic exposure, concomitant medications, SEC dosing (including the basis for dose selection and any dose adjustments during treatment), ASDAS and Bath Ankylosing Spondylitis Disease Activity Index (BASDAI) before and after at least 3 months of SEC therapy. Collected discontinuation events include stop, switch therapy, and loss to follow-up.”Stop” is defined as ≥90 days without a SEC prescription.”Switch therapy” is defined as switching to another treatment within 90 days after the last SEC prescription. Reasons for treatment discontinuation were also recorded.

### Statistical Analysis

Missing data were low for most variables (< 10%), except BASDAI (15.03%) and BMI (15.68%), which were handled using multiple imputation (MICE, 10 datasets, MAR assumption). All subsequent analyses were performed across the imputed datasets, and results were pooled according to Rubin’s rules. Descriptive statistics were utilized, presenting categorical variables as frequencies (%) and continuous variables as means (standard deviations) or medians (interquartile ranges, IQR). Differences between biologicsnaïve and biologic-experienced patients were assessed using the *chi-square* test or Fisher’s exact test for categorical variables, and the *t*-test or Wilcoxon rank-sum test for continuous variables, depending on data distribution. Persistence estimates were derived using Kaplan-Meier survival analysis, with treatment discontinuations as failure events. Differences between cohorts were compared *via* the log-rank test. Factors associated with drug retention were identified using univariate Cox regression. The following variables were included in the subsequent multivariate Cox proportional hazards model: Those with a *P*-value < 0.1 in the univariate analysis. Pre-specified clinically relevant variables: prior biologic therapy, dosage, disease duration, ESR, CRP, and BASDAI. The final model was refined using a backward stepwise approach to mitigate confounding bias. Results are reported as adjusted hazard ratios (HRs) and 95% confidence intervals (CIs). Statistical significance was defined as a two-tailed *P*-value < 0.05. All analyses were performed using SPSS version 26.0.

## Result

### Patient Characteristics

A total of 459 axSpA patients were included in this study, with a median age of 34 years (IQR: 29–41) and a median disease duration of 8 years (IQR: 5–13). The cohort was predominantly male (77.6%), and 92.2% were HLA-B27 positive. Over half of patients had prior exposure to biologics, primarily TNF inhibitors (TNFi) (56.2%). The median duration of SEC treatment was 9 months (IQR: 4–17). Patients with prior biologic experience were more likely to be concurrently using Nonsteroidal Anti-Inflammatory Drugs (NSAIDs) (23.1%), sulfasalazine (SSZ) (7.6%) and methotrexate (MTX) (1.3%). Inflammatory markers indicated with a median ESR of 19 mm/h (IQR: 7–23) and a median CRP of 8.51 mg/L (IQR: 1.91–10.10) at baseline. The median baseline BASDAI was 3.82 (IQR: 2.13–4.25), and the ASDAS score was 2.25 (IQR: 1.82–2.80). Radiographic assessment revealed that 88.7% of patients had radiographic axSpA (r-axSpA), while 11.3% had non-radiographic axSpA (nr-axSpA). Comparisons between biologics-naïve and biologic-experienced patients demonstrated significant differences in disease duration (*P* = 0.000), prior biologic use (*P* = 0.00), IL-17i use (*P* = 0.038), MTX use (*P* = 0.038), NSAID use (*P* = 0.005), ESR (*P* = 0.004), CRP (*P* = 0.009), and BASDAI (*P* = 0.042). A total of 42 patients (9.15% of the total cohort) received the 300 mg dosage treatment. Among these, 3 patients initiated treatment at 300 mg (1 due to coexisting PsO, and 2 starting at 300 mg at an external hospital for unknown reasons). Furthermore, 39 patients underwent dose escalation to 300 mg during the treatment period (Table 1).

**Table 1:** Demographic and clinical features of patients enrolled in the study

Characteristics		All (*n* = 459)	Biologics naive (*n* = 200)	Biologics experienced (*n* = 259)	*P* value
Age — median (IQR), years		34 (29, 41)	33 (29, 41)	35 (30, 41)	0.36
Male sex (%)		356 (77.56)	158 (79.0)	198 (76.45)	0.52
Disease duration (yrs)		8 (5, 13)	7 (3, 12)	9 (6, 14)	0.00
HLA-B27 (%)		423 (92.15)	184 (92.0)	239 (92.22)	0.91
		55 (11.98)	27 (13.5)	29 (11.19)	0.46
BMI		23.41 (21.36, 25.76)	22.91 (21.35, 25.71)	23.46 (21.41, 25.87)	0.66
SEC duration (mo)		9 (4, 17)	8 (4, 18)	9 (3, 17)	0.58
Biologics experience	TNFi (%)	258 (56.20)	0 (0.00)	258 (99.61)	0.00
	IL-17i (%)	5 (1.08)	0 (0.00)	5 (1.93)	0.07
	JAKi (%)	6 (1.30)	0 (0.00)	6 (2.31)	0.04
Concomitant treatment	SSZ (%)	34 (7.64)	16 (8.04)	18 (7.31)	0.67
	MTX (%)	6 (1.30)	0 (0.00)	6 (2.31)	0.04
	NSAIDs (%)	106 (23.09)	36 (18.00)	70 (27.02)	0.01
EMMs	UV (%)	27 (5.88)	10 (5.00)	17 (6.56)	0.48
	IBD (%)	4 (0.87)	2 (1.00)	2 (0.77)	0.80
	PsO (%)	12 (2.61)	4 (2.00)	8 (3.09)	0.67
Co-morbidity	HTN (%)	15 (3.27)	5 (2.50)	10 (3.86)	0.58
	DM (%)	2 (0.44)	0 (0.00)	2 (0.77)	0.51
	CAD (%)	2 (0.44)	2 (1.00)	0 (0.00)	0.19
Number of previous biologics (%)^*^		259 (56.4)	-	-	-
1 (%)		158 (34.4)	-	-	-
2 (%)		82 (17.86)	-	-	-
3 (%)		19 (4.14)	-	-	-
Dosage 300 mg (%)^#^		42 (9.15)	17 (40.47)	25 (59.52)	-
ESR (mm/h)		19.00 (7, 23)	20.00 (6, 34)	8.50 (5, 17)	0.00
CRP (mg/L)		8.51 (1.91, 10.10)	8.15 (2.19, 16.87)	2.67 (0.10, 10)	0.01
BASDAI		3.28 (2.13, 4.25)	3.00 (1.92, 4.06)	3.34 (2.52, 4.36)	0.04
ASDAS		2.25 (1.82, 2.80)	2.30 (1.86, 3.08)	2.17 (1.68, 2.67)	0.21
Morning stiness		1.00 (0.00, 2.00)	1.00 (0.00, 2.00)	1.00 (0.00, 2.00)	0.58
Morning stiness time		10.00 (0.00, 20.00)	10.00 (0.00, 20.00)	10.00 (0.00, 30.00)	0.47
SIJ radiographic status	nr-axSpA	52 (11.30)	23 (11.50)	29 (11.20)	0.92
	r-axSpA (%)	407 (88.70)	177 (88.50)	230 (88.80)	0.92

* If a patient uses both a biologic targeting the same pathway and its biosimilar, these are counted as two separate biologic experiences. However, the original drug and its generic are considered as one biologic experience. ^#^Including those who received a 300 mg dose either initially or after dose escalation during treatment. HLA, human leukocyte antigen; BMI, body mass index; SEC, secukinumab; TNFi, tumor necrosis factor inhibitor; IL-17i, interleukin-17 inhibitor; JAKi, Janus kinase inhibitor; SSZ, Sulfasalazine; MTX, methotrexate; NASAIDs, nonsteroidal antiinflammatory drugs; UV, urticarial vasculitis; IBD, inflammatory bowel diseases; PsO, Psoriasis; ESR, erythrocyte sedimentation rate; CRP, C-reactive protein; BASDAI, bath ankylosing spondylitis disease activity index; ASDAS, ankylosing spondylitis disease activity score. EMMs, extra-musculoskeletal manifestations; HTN, hypertension; DM, diabetes mellitus; CAD, coronary artery disease.

### Retention Rate and Predict Factors

**Figure 2:**
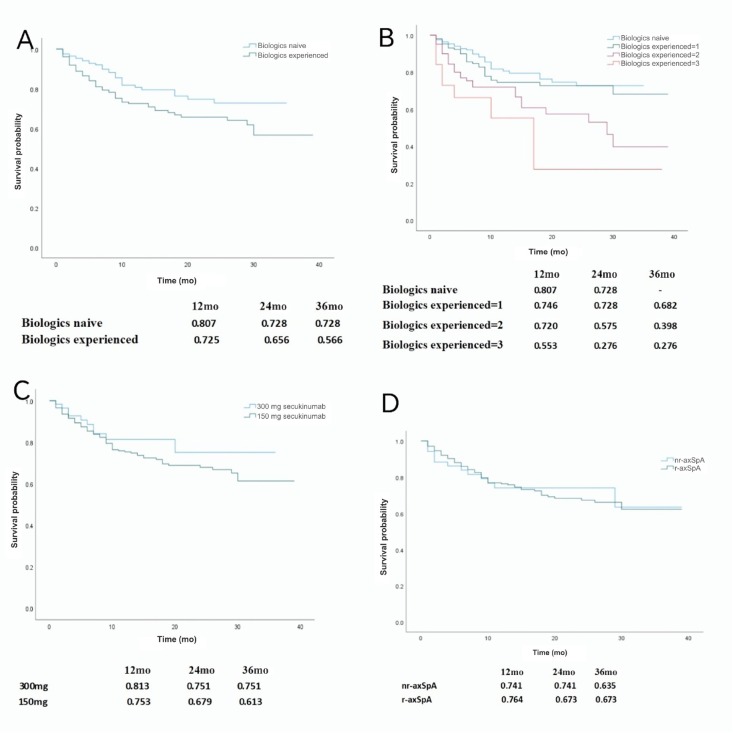
A: The Cox regression analysis curves for biologics experienced and biologics naive. B: The Cox regression survival curves, a based on the number of previous biologics experienced (naive, experienced = 1, 2, and 3). C: The Cox regression survival curves for different dosages of SEC (300 mg vs. 150 mg). D: The Cox regression survival curves for different dosages of nr-axSpA and r-axSpA.

Kaplan-Meier analysis determined the overall SEC retention rates to be 76.4%, 68.2%, and 62.8% at 12, 24, and 36 months, respectively. Biologics-naïve patients exhibited consistently superior retention compared to biologic-experienced patients (Figure 2A). At 12 and 36 months, rates were 80.7% and 72.8% for the naïve group versus 72.5% and 56.6% for the experienced group. Retention rates among biologic-experienced patients inversely correlated with the number of prior biologics used (Figure 2B). For patients with one, two, or three prior biologics, 12-month retention was 74.6%, 72.0%, and 55.3%, declining to 68.2%, 39.8%, and 27.6% by 36 months. A non-significant dose-dependent trend was observed, with the 300 mg group showing higher retention than the 150 mg group throughout follow-up (Figure 2C, *P* = 0.334). The 300 mg group’s rates were 81.3%, 75.1%, and 75.1% at 12, 24, and 36 months, respectively, compared to 75.3%, 67.9%, and 61.3% for the 150 mg group. Drug retention was comparable between disease subgroups (nr-axSpA *vs*. r-axSpA/AS), as evidenced by closely aligned Kaplan-Meier curves (Figure 1D). The 12-month retention rates were 76.4% (nr-axSpA) and 74.1% (r-axSpA), and the 36-month rates were 63.5% and 67.3%, respectively, indicating comparatively stable long-term retention across the entities (Figure 2D).

The multivariate Cox regression model, which was adjusted for potential confounding factors including disease duration, ESR, CRP, and BASDAI, showed that prior biologic therapy history remained an independent predictor of drug discontinuation. Prior biologic therapy history (biologics-experienced *vs*. naive) was significantly associated with a higher risk of discontinuation (adjusted HR = 1.68, 95% CI = 1.09–2.61, *P* = 0.020). Disease duration (adjusted HR = 0.98, *P* = 0.257), ESR (HR = 1.010, *P* = 0.561), CRP (HR = 0.99, *P* = 0.226), and BASDAI (HR = 1.04, *P* = 0.607) were not found to be independently associated with SEC retention in the final adjusted model (Table 2).

**Table 2:** The real-world studies comparing retention rates and switching patterns across different regions

Variable	Univariate HR (95% CI), *P*-value	Multivariate HR (95% CI), *P*-value
Biologics-experienced (*vs*. naive)	1.68 (1.11–2.56), *P* = 0.015	1.68 (1.09–2.61), *P* = 0.020
Disease duration (yrs)	0.99 (0.96–1.02), *P* = 0.490	0.98 (0.95–1.02), *P* = 0.257
ESR (mm/h)	0.99 (0.98–1.01), *P* = 0.700	1.010 (0.99–1.02), *P* = 0.561
CRP (mg/L)	0.99 (0.98–1.01), *P* = 0.197	0.99 (0.97–1.01), *P* = 0.226
BASDAI	1.06 (0.93–1.21), *P* = 0.374	1.04 (0.91–1.18), *P* = 0.607
nr-axSpA (*vs*. r-axSpA)	1.05 (0.58–1.88), *P* = 0.881	1.16 (0.64–2.12), *P* = 0.624

ESR, erythrocyte sedimentation rate; CRP, C-reactive protein; BASDAI, bath ankylosing spondylitis disease activity index.

### Discontinuation and Adverse Events

SEC was discontinued in 82 out of 459 patients (17.86%). The reasons for discontinuation included self-discontinuation (*n* = 8, 9.76%), cessation due to surgery (*n* = 2, 2.44%), discontinuation due to pregnancy planning (*n* = 2, 2.44%). 70 (85.37%) patients switched medications. Among those who switched, 31 (37.80%) transitioned to adalimumab (ADA) /biosimilar, 18 (21.95%) to etanercept (ETN) /biosimilar, 4 (4.88%) to Janus Kinase inhibitor (JAKi), 4 (4.88%) to Golimumab, 1 (1.22%) to Certolizumab pegol, and 2 to Ixekizumab. Additionally, 8 patients (9.76%) switched to conventional synthetic Disease-Modifying Antirheumatic Drugs (csDMARDs), such as SSZ and MTX, or on-demand NSAIDs. Reasons for switching included lack of efficacy and adverse reactions. Within our cohort, 4 patients had a history of IBD, with one transitioning to ADA due to IBD exacerbation during SEC treatment.^[7]^ Additional factors included economic constraints and pregnancy planning. Four patients developed injection site rashes, one experienced eczema, and one had oral ulcers, but these did not lead to discontinuation.

## Discussion

This retrospective multicenter real-world study evaluated the retention rate of SEC in an Asian population, offering valuable insights into the factors associated with long-term treatment persistence and discontinuation patterns. The overall retention rates at 12, 24, and 36 months were 76.1%, 68.7%, and 62.8%, respectively. A key finding is that retention rates were higher among biologics-naïve patients.

Previously reported retention rates of SEC in real-world settings have varied from 55% to 98% across different populations, including those with spondyloarthritis (SpA) and PsO (Table 3). An analysis showed that Poland and certain European countries, particularly the UK, exhibit significantly higher one-year drug retention rates compared to other nations. Specifically, Poland’s AS population reported a retention rate of 98%,^[4]^ while European countries averaged 85.5%,^[8]^ and the UK achieved 89.2% for r-axSpA populations.^[9]^ Further investigation revealed that both the European and UK cohorts were part of the “SERENA” study, where patients had already received 16 weeks of SEC treatment before enrollment. A similar phenomenon was observed in the Italian cohorts, where one-year survival rates reached 80%–90%. In the study by Gentileschi S, patients had already received 12 months of SEC treatment prior to inclusion, while Ramonda R’s study included those who continued taking SEC for more than three months. These inclusion criteria led to higher overall retention rates, highlighting the challenges in comparing outcomes across studies. In our study, axSpA patients with at least two prescriptions record of SEC were included, impacting retention outcomes. In analyzing the Polish population, it was found that 59.9% were biologics-naïve, compared to 43.6% in our cohort. This analysis, combined with the biologics-naïve proportions in other populations, supports our study’s conclusion that biologics-naïve patients have better drug retention rates. Overall, the analysis indicates that the Chinese population still shows significantly higher drug retention rates compared to other countries.

**Table 3:** The real-world studies comparing retention rates and switching patterns across different regions

Year	Disease	Country	Sample Size	Dose of 300 mg (%)	Biologics naive (%)	Retention rate (%)			Discontinuation rate (%)
						12 mo	24 mo	36 mo	
2020^[31]^	axSpA	Italy	39	56.4	25.6	80-90	78.2	NA	17.9
2022^[32]^	axSpA	Italy	249	23.7	28.9	80-90	75	60-70	24.5
2023^[3]^	axSpA	France	906	9.7	8	59	NA	NA	13.4
2023^[33]^	axSpA	Canada	146	19	23.3	62.9	NA	NA	35.6
2023^[11]^	r-axSpA	Turkey	147	0	15.6	55	NA	NA	42
2024^[34]^	axSpA	Italy	272	25	30.9	80-90	70-80	60-70	38.2
2024^[35]^	axSpA	Romania	46	30.4	8.7	59.7	31.3	20	41.3
2024^[23]^	AS	Turkey	166	NA	28.3	62	10-20	NA	56
2024^[30]^	AS	Israeli	38	NA	0.4	75.7	65-70	55-60	30.8
2025^[36]^	AS	Greek	81	NA	43.2	89.9	80.5	77.3	20.98
2025^[36]^	PsA	Greek	214	NA	26.6	87.1	76.9	74	24.77
2021^[22]^	axSpA/PsA	Spain	138	12/54	34	75	66	64	32.6
2021^[10]^	axSpA/PsA	Spain	154	12/57	8	66	43	NA	41.6
2022^[8]^	PsA/r-axSpA	European	1004	NA	NA	85.2/85.8	74.9/78.9	NA	7.3
2023^[9]^	PsA/r-axSpA	UK	189	56.6/3.8	43.4	91/89.2	77.6/76.2	NA	12.7
2022^[4]^	PsA/AS	Poland	187	48.7/10.5	59.9	91/98	85-90	NA	NA
2020^[37]^	PsA	Greece	75	100	53	66	56	NA	36
2021^[38]^	PsA	Italy	608	54.9	37.3	80-90	71	NA	20.23
2022^[12]^	PsA	European	2017	NA	21.9	76	NA	NA	38.5
2024^[39]^	PsA	Italy	207	76	51.1	80-90	65-75	60-70	23.7
2020^[40]^	PsO/PsA	Germany	68	100	58.8	68	50-60	NA	23.5
2022^[41]^	PsO	European	1756	100	65.9	88	76.4	60.5	10.1
2020^[13]^	PsO	UK	991	NA	72.9	88	77	NA	13.6
2023^[42]^	PsO	Vietnam	232	100	95.3	84.9	64.7	53.2	15.1
2023^[43]^	PsO	Japan	123	NA	47.2	78^#^	63.2	47.2	64.2

# 52-week drug retention rate. The data providing only the range of retention rates in the table is sourced from the images included in the original article. PsA, psoriatic arthritis; PsO, psoriasis; AS, ankylosing spondylitis; NA, not available.

Regarding predictors of drug retention, our study found that biologics-naïve patients had a higher likelihood of continuing SEC therapy, consistent with other studies, suggesting that initiating SEC as a first-line biologic therapy is associated with better long-term adherence and potentially improved clinical outcomes.^[3, 10, 11, 12, 13]^ Conversely, while other studies have identified male sex, obesity, smoking, achilles enthesitis, fibromyal-gia (FM) and peripheral phenotype as risk factors associated with SEC discontinuation, this trend was not observed in our cohort.^[14, 15, 16, 17, 18, 19]^

The optimal timing and criteria for SEC dose escalation represent a continuing clinical debate, as no clear guidelines currently define the cutoff for increasing the dose to 300 mg. Existing studies utilize varying thresholds: some define the escalation cutoff as an ASDAS score ≥ 1.3 after three months of 150 mg treatment,^[20]^ while others set a higher bar at ASDAS ≥ 2.1.^[21]^ In our study, the principle for dose escalation was the less stringent ASDAS≥ 1.3 after three months on 150 mg, and this relatively lower escalation threshold may potentially contribute to the higher drug retention rate observed in our cohort. The impact of dosage itself is a critical point of interest. Controversy persists regarding whether dose escalation is associated with higher retention rates. In our cohort, although no statistically significant difference in retention rate was observed between the 300 mg and 150 mg groups (*P* = 0.334), the 300 mg group demonstrated a numerical advantage over the lower dose. This finding aligns partially with a recent multicenter study, which reported that escalating the SEC dose to 300 mg after 150 mg treatment was associated with sustained improvement in disease activity, as well as favorable retention and safety profiles in patients with active ax-SpA. Conversely, in the Italian PsA population, 150 mg users were suggested to have better retention, while another study (Ruscitti *et al.)* found that dose differences were not predictive of discontinuation.^[22,23]^

A total of 82 patients (17.86%) discontinued SEC during the follow-up period, consistent with findings from other real-world studies (Table 2). Across these studies, the primary reason for discontinuation was either primary or secondary inefficacy. In our study, one case of IBD was observed. Other adverse events reported included injection site rashes, eczema, and oral ulcers. No serious adverse events, such as death or malignancy, were observed. Of those who discontinued, 70 patients switched to alternative therapies. Regarding switching patterns, our findings reveal that TNFi were the most common choice (65.85%), followed by csDMARDs or on-demand NSAIDs (9.75%), and JAKi (7.32%). Currently, no specific guidelines exist for switching therapies following biologic treatment failure. This study provides valuable real-world data on switching strategies after SEC failure in the Chinese population. We established a 90-day period for all discontinuation events, which is considered a “grace period.” This aligns with current literature, where a grace period of 90 days is applied for dosing intervals of ≤ 30 days, and 180 days for intervals greater than 30 days.^[13, 24, 25, 26, 27, 28, 29]^ However, in the Israeli AS population, treatment discontinuation is defined as the first gap of 120 days or more after the last supply date. With only 0.4% of patients being biologic-naïve, their 12-month retention rate reached 75.7%,^[30]^ higher than in most countries (Table 2). It is reasonable to speculate that a longer grace period may result in higher retention rates. Unfortunately, real-world studies in other countries have not provided detailed descriptions of the grace period for discontinuation, making it difficult to confirm this hypothesis. A more standardized definition of the grace period is needed.

We observed a discrepancy between baseline BASDAI and ASDAS scores: There was a statistically significant difference in baseline BASDAI between the biologics-naïve and biologic-experienced groups (*P* = 0.042), while the ASDAS score showed no significant difference (*P* = 0.21). Possible explanations for this discrepancy are: ASDAS incorporates objective inflammatory markers (CRP/ESR), whereas BASDAI is purely based on patient subjective report. Biologic-experienced patients may have altered subjective symptom perception or co-existing conditions such as fibromyalgia, leading to the observed discordance between BASDAI and ASDAS. Furthermore, some patients in our cohort were transferred from external centers or Hong Kong while already on SEC, and their baseline disease activity (not active status) was recorded at their first visit to our center. This transfer situation may also contribute to the observed difference between BASDAI and ASDAS.

The study’s strengths include its large sample size and the real-world setting, which enhances the generalizability of the findings. However, several limitations of our study should be noted. The retrospective design of this study introduces potential selection bias. As data were primarily sourced from the HIS, the cohort may be overrepresented by patients with better adherence who regularly visited our specialized center. Furthermore, reliance on electronic medical records inevitably led to incomplete data, particularly for baseline disease activity measures like the BASDAI score. This missing data may have resulted in the unintended inclusion of patients with milder baseline disease activity, potentially overestimating the overall drug retention rate. Moreover, well-established predictive factors, such as FM, could not be adequately analyzed due to the constraints of the study design. The inability to account for these variables limits a more comprehensive interpretation of the factors influencing SEC retention. Future prospective studies with more comprehensive data collection are needed to confirm our findings and further explore the factors influencing SEC retention in different populations.

In summary, this study provides important real-world evidence on SEC retention rates in Chinese axSpA patients, indicating good long-term adherence, particularly among biologics-naïve patients. This study provides real-world data on switching strategies following SEC failure, which may inform clinical practice in the Chinese population. Given the retrospective design of this study, conclusions must be interpreted with caution and require further validation in larger, prospective studies.
